# Are the Gut Bacteria Telling Us to Eat or Not to Eat? Reviewing the Role of Gut Microbiota in the Etiology, Disease Progression and Treatment of Eating Disorders

**DOI:** 10.3390/nu9060602

**Published:** 2017-06-14

**Authors:** Yan Y. Lam, Sarah Maguire, Talia Palacios, Ian D. Caterson

**Affiliations:** 1 Boden Institute of Obesity, Nutrition, Exercise & Eating Disorders, Charles Perkins Centre, University of Sydney, Sydney, NSW 2006, Australia; sarah.maguire@sydney.edu.au (S.M.); talia.palacios@sydney.edu.au (T.P.); ian.caterson@sydney.edu.au (I.D.C.); 2 Department of Biochemistry and Microbiology, School of Environmental and Biological Sciences, Rutgers, The State University of New Jersey, New Brunswick, NJ 08901, USA

**Keywords:** gut microbiota, eating disorders, appetite control, psychological stress

## Abstract

Traditionally recognized as mental illnesses, eating disorders are increasingly appreciated to be biologically-driven. There is a growing body of literature that implicates a role of the gut microbiota in the etiology and progression of these conditions. Gut bacteria may act on the gut–brain axis to alter appetite control and brain function as part of the genesis of eating disorders. As the illnesses progress, extreme feeding patterns and psychological stress potentially feed back to the gut ecosystem that can further compromise physiological, cognitive, and social functioning. Given the established causality between dysbiosis and metabolic diseases, an altered gut microbial profile is likely to play a role in the co-morbidities of eating disorders with altered immune function, short-chain fatty acid production, and the gut barrier being the key mechanistic links. Understanding the role of the gut ecosystem in the pathophysiology of eating disorders will provide critical insights into improving current treatments and developing novel microbiome-based interventions that will benefit patients with eating disorders.

## 1. Introduction

Eating disorders are severe mental illnesses that occur on a continuum with behaviors shared across syndromes that negatively influence cognitive, physiological, and social functioning. The prevalence of eating disorder behaviors in the community is on the rise, with a cross-sectional general population survey in South Australia reporting a doubling of prevalence in adults to 8.4% over a decade, and the demographic profile deviated from predominantly young white upper-class women to an increase in men and those in older age groups and those of lower socioeconomic status [1,2]. Although onset could happen at any time across the lifespan, the majority of eating disorders begin during adolescence and early adulthood. One study in a large US city reported that 13% of young women experienced at least one eating disorder by age 20 [3]. While most point to negative body image and/or concerns with body weight as the primary etiology of eating disorders (and thus they are classified as mental illnesses), evidence for disturbed appetitive and feeding pathways suggest that eating disorders may also be biologically-driven. Whatever the cause(s) may be, it typically leads to controlled eating, and when this pursuit becomes an obsessive focus in life, patients pursue extreme dietary restriction, binge eating, and compensatory behaviors. From there on, mood disturbance and metabolic dysfunctions further contribute to physical and psychosocial morbidity. Reduced income and employment, heavy carer burden, and elevated health care cost see eating disorders not only impact negatively on an individual but also on a societal level [4,5,6,7].

It is now clear that the gut microbiota is necessary for normal physiology, and that a state of dysbiosis (a microbial profile that deviates from that found in healthy individuals) increases the risk of diseases. The growing body of literature on the effects of the gut microbiota on host health, ranging from nutrient/energy metabolism to brain function, led us consider a role of this “forgotten organ” in the etiology and pathophysiology of eating disorders. Given the established gut–brain and gut–diet interactions, the gut microbiota may well be the critical mechanistic link between psychological and biological factors in these illnesses. More importantly, compensatory behaviors, e.g., purging and laxative abuse, and conventional treatment (nutritional rehabilitation) would be expected to impact on the gut microbiota and this change may feed back to modify the disease progress further. Here we review the evidence for the gut microbiota as an integral part of eating disorders, from onset to progression and treatment. We propose new research to increase our understanding and then possibly harness the therapeutic potential of the gut bacteria to improve the outcome of eating disorders.

## 2. Overview of Eating Disorders: Classic Etiology, Progression, and Treatment

The classification “eating disorders” describes a group of mental illnesses that manifest with disturbance to feeding behaviors and body weight regulation, with subsequent compromise across key physiological systems including gastrointestinal and cardiovascular functions. The fifth edition of the Diagnostic and Statistical manual from the American Psychiatric Association [8] recognizes three primary diagnoses within the eating disorder category: anorexia nervosa (AN), bulimia nervosa (BN), and binge eating disorder (BED) (Table 1). Eating disorder presentations that do not fit within these diagnoses (approximately 20–40% of cases) are classified under residual categories (Other Specified or Unspecified Feeding or Eating Disorders; OSFED). Although diagnostic distinctions are made between the categories, a number of symptoms (e.g., caloric restriction, purging, binging, over-evaluation of body weight/shape) are shared across diagnoses of eating disorders (Table 1).

The exact etiology of eating disorders is unknown, although genetic and neurobiological predispositions are emerging as important, and are believed to interface with environmental and socio-cultural influences, as well as psychological traits, to cause illness. Relatives of a person with an eating disorder are 7–12 times more likely to develop the illness themselves [9,10,11]. The role of genetics is further supported by data from twin studies that estimate heritability accounts for 30–80% of AN and BN [10,12]. Importantly, age and pubertal maturation appear to contribute to the emergence of genetic risk for disordered eating symptoms during mid-to-late adolescence and puberty, possibly due to sexual maturity (physical appearance and hormonal changes) and increased cultural pressure for the thin ideal [13,14,15].

There are advocates for a neurobiological origin of eating disorders, particularly regarding the role of the hypothalamus in appetite and body weight control. Neuropeptide and neuroendocrine dysregulation is typical of eating disorders [16] and functional magnetic resonance imaging (fMRI) studies revealed an altered set-point and/or sensitivity for sensory-interoceptive-reward processes towards food consumption that may override homeostatic needs [17]. What remains unclear, however, is whether patients with eating disorders have a primary disturbance of neurobiology or whether this is merely a consequence of physiological alterations caused by the disease process. Finally, psychological and psychosocial traits are universally recognized as key components in the etiology of eating disorders. Impaired psychosocial functioning, perfectionism, thin-ideal internalization, negative urgency, and sensitivity to reward and punishment are among the key risk factors that may predispose people to the onset of these illnesses [18,19].

Beyond etiology, eating disorders are further complicated by their instability and chronicity—the illnesses can quickly progress from being active to recovery and relapse, and patients typically undergo repeated relapsing-remitting courses and even transitions from symptoms of anorexia to bulimia, and vice versa, throughout the lifespan [20]. While some complications are a direct consequence of the disordered feeding behaviors, e.g., vomiting and laxative abuse leads to electrolyte disturbance, others are primarily due to poor nutritional intake, notably deranged gastric motility, constipation, and reduced bone mineral density [21,22]. These complications not only compromise physiological functions, but also trigger psychological stress. The resulting distress, depression, and anxiety then further contribute to the vicious spiral of long-term morbidities. To date, the treatment for eating disorders typically consists of a combination of the management of medical complications, psychosocial/psychiatric therapy, and nutritional rehabilitation. Interventions that are tailored to the individual patient’s clinical and psychopathological features, as well as their response to previous treatments, are generally considered the most likely to improve the outcome for eating disorders [23,24].

## 3. Gut Microbiota: A Missing Piece in Eating Disorders

### 3.1. Dysbiosis in Eating Disorders

Each person has a unique and yet highly dynamic gut ecosystem that depends on complex interactions between genetic and environmental factors. Similarities in the microbial composition and functions among healthy individuals are suggestive of a core microbiome that is required for host health [25]. Many diseases, ranging from metabolic (e.g., obesity and type 2 diabetes) to autoimmune (e.g., multiple sclerosis) and neurodegenerative (e.g., Alzheimer’s disease), have now been linked to dysbiosis [26,27,28] and extensive research efforts have gone into developing treatments to achieve a healthy microbiome. Given that host diet is a key determinant of the gut microbial profile and eating disorders are characterized by dysregulated food intake, it is only logical to assume at least an associative relationship between eating disorders and an altered gut microbiota. Surprisingly though, literature on this area is scarce, with only a handful of studies measuring gut microbial profile in patients with AN [29,30,31,32] and no data are available on other forms of eating disorders.

### 3.2. The Microbiota-Gut-Brain Axis

The gut–brain axis, connected via neural, hormonal, and immunological pathways, is a bi-directional communication system that is initially recognized for its role in regulating digestive function and food intake [33,34]. There is a high prevalence of co-morbidity between psychiatric and gastrointestinal symptoms, e.g., 40–60% of patients with functional gastrointestinal disorders experience psychiatric symptoms [35] and up to 50% of psychiatric patients are diagnosed with irritable bowel syndrome [36], which clearly suggests broader implications of this axis on gastrointestinal and brain functions. Recent advances in our knowledge of the gut microbiota have shed new light on the interactions between the brain and the gastrointestinal tract, with microbiota now being considered an integral part of the gut–brain communication—some even advocate for microbiota being an independent component of the axis [37,38].

#### 3.2.1. Effects of Gut Microbiota on Appetite Control

Altered gut–brain communication is evident in eating disorders with dysregulated appetite control and a distorted perception of satiety among the key biological drivers of extreme feeding behaviors. From an evolutionary perspective, it is not difficult to envisage a role of gut microbiota in modifying host feeding behavior as different bacteria have distinct nutritional requirements, e.g., *Prevotella* thrive on carbohydrate and *Bacteroides* appear to have a preference for protein and animal fat [39]. Alcock and colleagues [40] further hypothesized that the diversity of the microbial population is the key to how gut microbiota regulate host food intake, as dominance of any particular microbial groups would impose greater selective pressure, and thus a positive feedback loop, on the host that potentially leads to particular dietary preferences and/or patterns.

There are no definitive studies on such a microbiota-host food intake relationship in humans, but animal data point to a few plausible mechanisms. The first is the impact of gut bacteria on the production and/or activity of appetite-regulating hormones. Enteroendocrine cells express Toll-like receptors which, when activated by binding with bacterial products (e.g., lipopolysaccharides (LPS) and flagellin), modify the secretion of hormones (such as cholecystokinin) that regulate satiety and hunger [38]. There is also evidence for the gut bacteria, primarily via LPS production, to modulate the effects of the central nervous system on gastrointestinal function, food intake, and energy homeostasis. LPS disrupts the blood–brain barrier (i.e., increases permeability) [41] to increase the impact of circulating cytokines on central appetite regulation; some animal data suggest that LPS directly initiates an anorexic response (i.e., inflammation-induced anorexia) by activating the Toll-like receptor 4/MyD88 signaling pathway in the central nervous system [42,43], although it has also been proven otherwise [44].

Another key mechanism by which gut bacteria influence food intake is by producing peptides that are sequence analogues of mammalian appetite-regulating hormones. These peptides then mimic the effect of the host’s hormones, and/or trigger an autoimmune response that interferes with normal appetite regulation, i.e., the host produces antibodies against the microbial peptides, which also act as autoantibodies that counteract the effect of the host’s own hormones [40]. The latter may be particularly relevant to the pathogenesis and progression of eating disorders, as Fetissov and colleagues [45,46] revealed that a subgroup of patients with AN and BN had autoantibodies that bind to the α-melanocyte-stimulating hormone (MSH), and the circulating level of these autoantibodies was correlated with the psychological traits of eating disorders. Finally, data on the bacterial protein C1pB (produced by both commensal and pathogenic microorganisms) as a mimetic of α-MSH and its effects on activating host satiety pathways in rodents [47], as well as the elevated plasma concentration of anti-C1pB IgG that is cross-reactive with α-MSH in patients with eating disorders [48], accord with the notion that autoantibody-induced interference with the central melanocortin system is one of the key microbiota–gut–brain mechanisms that contributes to dysregulated appetite control in eating disorders.

#### 3.2.2. Effects of Gut Microbiota on Brain Function and Behavior

We next explore the effects of microbiota on the gut–brain axis in the context of psychobehavioral abnormalities associated with eating disorders. Psychiatric and neurodevelopmental illnesses, including major depressive disorder [49,50], autism spectrum disorder [51], and multiple sclerosis [26], are consistently associated with a state of dysbiosis. Gut microbes are required for normal brain function. The behaviors of rodents with depleted gut microbiota (either were born and raised germ-free or were subjected to chronic antibiotics) exhibited impaired cognition and increased depressive-like behaviors [52,53]. Further, germ-free mice receiving cecal content transplant expressed behavioral phenotypes that mimicked the donors [54], and those that had fecal transplant from patients with major depressive disorder also displayed more depression-like behaviors, as compared to those colonized with microbiota from healthy controls [50]. These data suggest that behavioral traits are transmissible via gut microbes, thus providing strong evidence for causality between gut microbiota and psychobehavioral characteristics.

The effects of gut bacteria on behavior are primarily mediated by their actions on the hypothalamic–pituitary–adrenal (HPA) axis, a major neuroendocrine system that regulates the response to both psychological and physical stressors and is fundamental to the etiology and progression of eating disorders [55]. There appears to be a window early in life during which gut microbes are required for normal programming of the HPA axis. Colonization with a pathogen-free microbiome at the neonate stage reversed the exaggerated HPA stress response in germ-free mice but was without effect when the microbes were introduced later in life, which would implicate a role of neonatal dysbiosis (or infection) in predisposing life-long stress-related pathologies [56].

There is also evidence for gut microbiota to continue its effect on the HPA axis in adult life by modulating its activity via neural and cytokine-mediated pathways. Bacteria produce many neurotransmitters and neuromodulators, e.g., γ-amino butyric acid (GABA) from *Bifidobacterium* spp. controls anxiety and serotonin from *Enterococcus* spp. modulates mood regulation, that either act directly on afferent axons or interact with the intestinal epithelial cells and thus the enteric nervous system to modify neural signaling to the central nervous system [37]. LPS, a metabolite from Germ-negative bacteria, provides an alternative route for the gut microbiota to modulate behavioral and cognitive parameters. Peripheral administration of LPS has been shown to induce peripheral- and brain-mediated responses in both animals and humans that mimicked the effects of bacterial infection [57,58]. By binding to its receptors on macrophages, lymphocytes, and granulocytes, LPS induces cytokine production (notably interleukin-1, interleukin-6 and tumor necrosis factor-α) from immune cells and triggers a series of changes in the immune-endocrine-nervous system that subsequently activates the HPA axis (for details please refer to a comprehensive review by Tilders and colleagues [59]). Vedder et al. [60] further demonstrated that LPS was able to increase the activity of the HPA axis in healthy humans in a dose-dependent manner. Finally, although circulating LPS does not pass the blood–brain barrier to impact on neurons or glial cells per se [59], LPS modulates the blood–brain barrier function by its direct effect on the tight junction proteins and thus barrier permeability, as well as its interactions with the brain endothelial cells to regulate immune cell trafficking and cytokine transport as an indirect way for gut bacteria to influence the central nervous system [61].

#### 3.2.3. Effects of the Brain on Gut Microbiota

While extensive research efforts have gone into understanding how the gut microbes alter brain function, the other direction of the microbiota–gut–brain axis has received relatively little attention. One would expect the central and autonomic nervous systems to play a key role in gut bacterial colonization. By regulating gastrointestinal functions, e.g., motility, nutrient absorption, acid production and mucosal immunity, the brain shapes the gut habitat and therefore selects for a distinct microbial profile [62]. There is also evidence for direct interactions between gut microbes and the host signaling molecules that are released into the intestinal lumen. Known as inter-kingdom signaling, many microorganisms express receptors for eukaryotic hormonal signals that primarily act by regulating gene expression [63], e.g., when exposed to norepinephrine, *Campylobacter jejuni* exhibited increased growth and virulence-associated properties in vitro [64].

In the context of eating disorders, the effect of psychological stress is the most relevant example of how the illness alters the gut microbiota. Chronic social stress (e.g., social disruption by aggressive co-inhabitants) and early life stress (e.g., maternal separation) have been shown to alter the diversity and composition of the gut microbiota in rodents [65,66,67]. A study in infant rhesus monkeys further demonstrated an inverse relationship between stress-indicative behavior, the abundance of *Lactobacilli*, and that of total aerobic and facultatively anaerobic bacteria in fecal samples when they were separated from their mothers [68]. Stress-induced dysbiosis (and the subsequent impaired intestinal barrier and bacterial translocation) has been proposed as a key molecular mechanism that stimulates innate immune activity and contributes to the association between chronic psychological stressors and systemic inflammation in humans [69,70].

### 3.3. Effects of Eating Disorder-Related Behaviors on Gut Microbiota

Food restriction in patients with eating disorders changes energy substrate availability (type, amount, and duration) for the gut microbes and leads to distinct microbial profiles. Limited food choice is a direct selective pressure as different microbes have their preferred substrates, e.g., *Roseburia* and *Bacteroides* are sensitive to dietary carbohydrate and protein, respectively, and the proportion of Bacteroidetes is dependent on the type of dietary fiber that reaches the colon [71,72,73]. Data from various classes of vertebrate hosts revealed that during prolonged fasting, comparable to the case of AN, microbes that utilize host intestinal mucins are able to flourish in the absence/shortage of dietary nutrients [74,75]. Following this notion, the abundance of the mucin-degrading taxa *Verrucomicrobia* was significantly increased in AN patients at baseline and returned to levels similar to that in healthy controls after weight gain [32]. The bloom of *Methanobrevibacter*, a bacterial genus that generates methane from hydrogen and carbon dioxide, is another example that the gut ecosystem of AN patients increases energy harvest in response to low nutrient availability [31]. The collective effect of chronic caloric deprivation in individuals who suffer from AN [30] or malnourishment [76] appears to be a reduction in the diversity of gut microbial communities that is often associated with poor clinical outcomes. Importantly, animal data further suggest that this “fasting” microbiome also contributes to malnourishment in the host, as a microbial transplant from individuals with Kwashiorkor (a form of severe protein-energy malnutrition) induced weight loss and altered protein and carbohydrate metabolism in the recipient mice [77].

Intermittent periods of feeding and withholding food is typical in almost all classes of eating disorders. Irrespective of total caloric consumption, such an eating pattern also impacts on the gut microbiota. Gut microbes exhibit time-specific changes in composition and functions that align with the host circadian clock, with up to 10% of all microbes in humans shown to display diurnal oscillations that contribute to distinct functional entities throughout the day, e.g., energy metabolism pathways dominate during daytime and the detoxification pathways are most active at night [78]. Collectively, changes in the intestinal pH and nutrient and secondary metabolite availability are expected to play a role in host metabolism [79]. Time-restricted feeding in mice [79] and repeated prolonged cycles of feeding and withholding food in dogs [80] have all been shown to cause a change in the overall gut microbiome that appears to be driven by changes in the abundance of species that differ by their preferred fermentable substrates (e.g., host-vs. diet-derived glycans). Cycling of microbial abundance appears to align with the feeding schedule that overrides the effect of the host circadian rhythm, which would implicate a role of irregular feeding patterns in contributing to dysbiosis in eating disorders.

Finally, purging behaviors, including self-induced vomiting and laxative/diuretic abuse, are common among patients with eating disorders for weight control purposes, but their effects on the gut microbiome have rarely been explored. Two studies described the gut microbial community structure in the restrictive and the binge–purging type of AN patients. Morita et al. [29] found no significant difference between the two subtypes when the abundances of individual species were compared. Using constrained ordination techniques, Mack et al. [32] reported that the overall microbial structure (assessed using constrained ordination techniques) was different between the two AN subtypes, but no specific species that drove this difference were identified, and they further demonstrated a reduction in microbial diversity in those who reported using laxatives. The exact mechanisms by which these behaviors affect the gut microbes remain largely unclear, but are expected to be a consequence of structural and functional changes in the colon, e.g., damage to mucosal lining, electrolyte imbalance, as well as changes in transit time and intra-abdominal pressure, which collectively alter the gut environment for microbial colonization [81,82].

### 3.4. Effects of Nutritional Rehabilitation on Gut Microbiota

Nutritional rehabilitation, also known as refeeding, is a key component of eating disorder treatment that aims to restore physiological functions by reversing malnutrition. Efficient attainment of a healthy body weight has been shown to predict recovery and thus aggressive approaches including high caloric meals and/or enteral feeding are common, especially in institutionalized patients [83]. The low content (or even absence) of fiber and resistant starch in these feeding regimens limits energy substrate availability for the gut microbes; abnormal fluid secretion into the colon triggered by enteral formula infusion also confers deleterious effects on the gut microbiota [84,85]. An effect of enteral feeding on gut microbial colonization is evidenced by the reduction in total fecal bacteria and short-chain fatty acid content in healthy humans after two weeks of fiber-free enteral feeding [86]. There are some data on gut microbiota in patients with AN following institutionalized nutritional rehabilitation programs that suggest that refeeding increases gut microbial diversity and shifts the composition towards, but remains significantly different from that of healthy controls [30,32].

### 3.5. Mechanisms Underlying the Microbiota-Induced Dysmetabolism in Eating Disorders

Morbidity and mortality of eating disorders are not always a direct consequence of malnutrition and/or psychiatric symptoms, but are often due to the deleterious metabolic sequelae of the illness, e.g., an increased risk of developing type 2 diabetes [87]. The molecular mechanisms by which dysbiosis induces metabolic dysfunctions are well-documented [28] and it is logical to hypothesize a similar role of gut microbes in dysmetabolism associated with eating disorders.

#### 3.5.1. Immune Function

Modulation of the host immune function is fundamental in the microbe–host relationship. Patients with eating disorders have been shown to have a greater risk of developing autoimmune diseases or conditions with an auto-inflammatory etiology [88]. Gut microbiota modulate both the adaptive and innate immune systems of the host, which often leads to systemic outcomes that go beyond the site of colonization. At the molecular level, the selective binding of bacterial components and metabolites (e.g., flagellin and LPS) to pattern-recognition receptors and/or the direct adhesion of bacteria themselves to the gut epithelium triggers an antigen-specific response that stimulates the adaptive immune system [89]. How the microbiota modulate innate immunity is less clear as the system lacks antigen specificity, but is believed to respond broadly to the activity of microbes via tissue-level microbial sensing [90].

#### 3.5.2. Short-Chain Fatty Acid Production

Microbial fermentation is a key mechanism by which gut bacteria impact on host metabolism. Dietary fiber and undigested carbohydrates are the primary substrates for fermentation, with short-chain fatty acids (SCFAs), notably butyrate, acetate, and propionate, the key end-products generated in this process. Butyrate is locally consumed by colonocytes as an energy substrate that influences the physiology of the colon. Most SCFAs indirectly impact on host metabolism by functioning as signaling molecules, e.g., as inhibitors for histone deacetylases or ligands for G-protein-coupled receptors, in pathways that modulate functions ranging from inflammation to energy homeostasis [91,92,93]. SCFA production is dependent on the host feeding pattern and gut microbial composition. David et al. [94] showed that an animal-based diet shifted the gut microbial structure in humans such that the abundance of bile-tolerant microbes increased at the expense of those that metabolized dietary plant polysaccharides, an effect associated with concomitant changes in the proportion of SCFAs that originated from different macronutrients. Cross-feeding, i.e., microbes utilizing metabolic end-products from one another, is another way by which fermentable substrate availability selects for particular microbial profiles [95]. Altered SCFA production has been demonstrated in patients with AN, who, when compared to healthy controls, had similar total SCFA levels but a greater proportion of branched-chain fatty acids due to fermentation of an endogenous protein source (an alternative energy substrate in the presence of low carbohydrate and dietary fiber intake) [32]. These protein-derived SCFAs may be of particular relevance to metabolic dysfunctions in eating disorders, as isobutyrate, 2-methylbutrate, and isovalerate (that are exclusively generated from branched-chain amino acids) have been implicated in insulin resistance [96].

#### 3.5.3. Gut Barrier Function

Gut microbe-induced changes in gut permeability have been demonstrated in metabolic dysfunctions as a key mechanistic link between dysbiosis and systemic inflammation. An impaired gut barrier (or a “leaky” gut) increases the translocation of microbial metabolites, or even the microbes themselves, from the lumen of the gastrointestinal tract into the adjacent tissues. These materials then enter the portal and systemic circulation, a phenomenon known as endotoxemia, to trigger inflammatory responses at the tissue level [97]. In obese rodents, antibiotics and prebiotics were able to restore gut barrier integrity and metabolic functions, providing evidence for the gut barrier as a mediator of microbiota-induced systemic inflammation [98,99]. Gut microbes do not directly disrupt the gut barrier, rather they alter butyrate availability for colonocytes that change colonic mucosal functions [100], and they induce the secretion of pro-inflammatory cytokines that disrupt tight junction proteins [101]. Interestingly, there appears to be a region-specific effect of eating disorders on gut permeability. Experimentally-induced AN increased barrier permeability and reduced tight junction protein expression in the mouse colon, but was without effect in the jejunum [102]. Colonic gut permeability data are not available in patients with eating disorders, but a study by Monteleone et al. [103] showed a significant decrease in small intestinal permeability in AN patients. The potential distinct effects of eating disorders on barrier function of different parts of the gut warrant further investigation, but a localized effect on the colon may provide further support for a predominant role of microbes (that primarily reside in the colon) in mediating gut dysfunction in eating disorders.

## 4. Therapeutic Potential of Microbiome-Based Treatments for Eating Disorders

**Figure 1:**
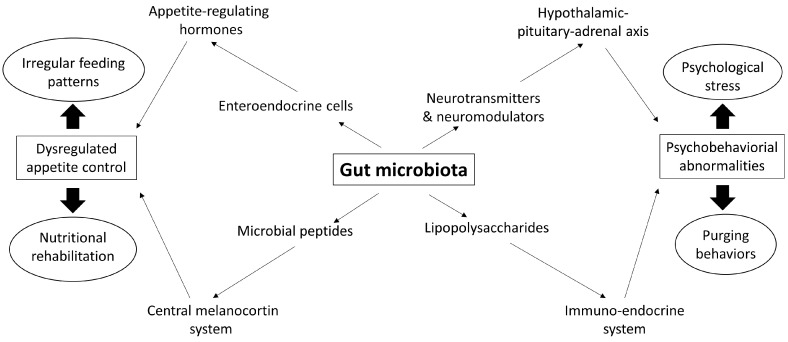
Potential mechanistic role of the gut microbiota in the etiology and progression of eating disorders. The gut microbiota has been shown to modulate the hypothalamic–pituitary–adrenal axis and the enteroendocrine, central melanocortin, and immune-endocrine systems that may collectively contribute to dysregulated appetite control and psychobehavioral abnormalities typically seen in eating disorders. Emerging evidence also suggests that the outcomes of the illnesses and the related treatments (in ovals) may feed back to the gut ecosystem that further negatively impact on the progression of the diseases.

There is a growing body of evidence suggesting that gut microbiota is mechanistically involved in changes in physiological function throughout the etiology, progression, and treatment of eating disorders (Figure 1). It is very tempting to jump straight on the bandwagon of using probiotic/prebiotic supplementation as the novel interventions for eating disorders, but a lot of work is still required before we get to the position where we are ready to develop microbiome-based treatments for these patients. Adapting the model currently used in elucidating the role of gut microbiota in metabolic diseases [104], the key steps are characterizing the gut microbiota in patients with eating orders, correlating the microbial characteristics to disease phenotypes, and potentially establishing causality. First, we need to distinguish the gut microbiota between different classes of eating disorders and have longitudinal cohorts with sufficient sample size and detailed repeated sampling to capture the variations in microbial profile and functions across vastly different individuals and over the course of diagnosis, disease progression, treatment, and recovery. These could then be mapped with physiological, psychological, and cognitive changes to identify bacteria that are of potential relevance to the disease. Thereafter, the candidate species/strains should be tested in pure cultures in vitro and in gnotobiotic animal models to understand the host–microbe biology and the underlying molecular mechanisms of causality. Only then are we able to determine when and how we should intervene and the efficacy of a gut microbiome-targeted treatment regimen for eating disorders.

While we are still a long way from using strategies that restore a healthy gut microbiome to treat eating disorders, we can already see the possibility of using microbiome-targeted interventions to supplement what is already available for the patients. For example, a major challenge for treating eating disorders is to engage patients in sustainable healthy eating patterns, as compliance to nutritional rehabilitation is often hindered by gastrointestinal symptoms associated with the illness, e.g., nausea, early satiety, post-prandial discomfort, abdominal pain, and constipation. Prebiotic and probiotic supplementations have already been shown to relieve symptoms and improve gut functions in patients with functional and inflammatory bowel disorders [105,106]. Achieving and maintaining a healthy gut microbiome, therefore, could potentially be a gut-specific approach to increase treatment efficacy in eating disorders. Following this notion, the positive effects of prebiotic supplementation on the gut microbial profile and quality of life in tube-fed patients [107] may implicate similar enteral feeding modifications in refeeding regimens for patients with eating disorders. Recent data on the psychological benefits of optimizing gut microbiota using probiotics, notably in reducing anxiety and depressive symptoms [108,109], further suggest another key aspect of eating disorders in which microbiome-based treatments may confer benefits.

## 5. Conclusions

Based on what we already know about the physiological functions of the gut microbiome in the context of metabolic diseases, and the predicted changes in the host–microbe biology in eating disorders, one could be confident that the gut microbiota is likely to play a key role in various stages of the illnesses. More work is needed in this relatively new area to further our understanding of how gut microbes impact the human host under the distinct set of circumstances in eating disorders. Specifically, generating high quality longitudinal metagenomic data that characterize the gut ecosystem structure and functions are the key short-term goals for two important purposes: (1) cohorts of high-risk individuals allow insights into the role of gut microbiota in the etiology of eating disorders; and (2) following cohorts of patients with eating disorders would allow advances in our understanding of how the gut microbiota changes with clinical observations and whether causality exists. Only when we identify core group(s) of bacteria or functional guilds that are potential causative agents, are we in the position of developing evidence-based microbiome-targeted treatments, as well as modifying current treatments to improve efficacy, to eventually benefit patients with eating disorders.

## Figures and Tables

**Table 1:** Diagnostic criteria and criterion behaviors for eating disorders.

Categories of Eating Disorders	Diagnostic Criteria (DSM-5) ^1^	Eating Disorder Behaviors					
		Restrict	Binge	Vomit	Laxative	Over-Exercise	Body, Weight & Shape Concerns
Anorexia Nervosa (AN)	Significant weight loss; fear of weight gain; body weight & shape concerns	_+_	_+/−_	_+/−_	_+/−_	_+/−_	_+_
Bulimia Nervosa (BN)	Regular binge eating; compensation behaviors (e.g., vomiting, laxative abuse); body weight & shape concerns	_+/−_	_+_	_+/−_	_+/−_	_+/−_	_+_
Binge Eating Disorder (BED)	Regular binge eating, at least 3 of 5 other negative features (e.g., eating large amounts when not hungry, eating alone due to embarrassment); significant distress	_+/−_	_+_				_+/−_

^1^ Fifth edition of the Diagnostic and Statistical manual from the American Psychiatric Association; _+_ Indicates must be present; _+/−_ Indicates can be present.
